# Attachment Style and Childhood Traumatic Experiences Moderate the Impact of Initial and Prolonged COVID-19 Pandemic: Mental Health Longitudinal Trajectories in a Sample of Italian Women

**DOI:** 10.1007/s11469-022-00798-x

**Published:** 2022-03-17

**Authors:** Giovanni Castellini, Livio Tarchi, Emanuele Cassioli, Eleonora Rossi, Giulia Sanfilippo, Matteo Innocenti, Veronica Gironi, Irene Scami, Valdo Ricca

**Affiliations:** 1 Psychiatry Unit, Department of Health Sciences, University of Florence, Florence, Italy; 2 Padiglione 8b, Cliniche Chirurgiche Primo Piano, AOU Careggi, Viale della Maternità, 50134 Firenze, FI Italy

**Keywords:** COVID-19, Psychopathology, Quarantine, Lockdown, Women, Pandemics

## Abstract

The impact of the COVID-19 pandemic on mental health has not been clarified yet, with multiple studies warranting a special focus on women and young adults. A sample of 101 Italian women recruited from the general population was evaluated a few weeks before the onset of the pandemic and during the first and the second wave of the pandemic. Depression values at the Brief Symptom Inventory showed an initial increase followed by a stabilization on higher values in respect to the baseline, whereas Phobic Anxiety was stably worsened. Participants with insecure attachment styles and childhood trauma showed higher levels of distress at all timepoints. In many psychopathological domains, moderation analysis showed an unfavorable trend over time for younger participants. The present study seems to confirm a high burden on mental health for women during the COVID-19 pandemic, highlighting young age, insecure attachment style, and childhood trauma as negative prognostic factors.

## Introduction

The emergence of COVID-19 has been posited to threaten both the short-term survival of many individuals and the long-lasting mental wellness in the general population (Loades et al., 2020; Xiong et al., 2020). In fact, an impact by the generalized and prolonged conditions of insecurity was observed in several cross-sectional examinations for what concerns early-affected communities, e.g., Northern Italy and Mainland China (Fiorillo & Gorwood, 2020; Gloster et al., 2020; Hossain et al., 2020). Discordant reports of worsened mental conditions during COVID-19 pandemic may be due to the high degree of heterogeneity in enrolled populations, in particular for vulnerable and understudied individuals, as in the case of young adults, children, adolescents, women, and elderly (Copeland et al., 2021; Gruber et al., 2021; Nearchou et al., 2020; Rettie & Daniels, 2021; Sepúlveda-Loyola et al., 2020). In Italy, multiple sources highlighted increased rates of anxiety, depression, and feelings of distress — especially among those vulnerable and understudied populations already mentioned (Delmastro & Zamariola, 2020). Indeed, worse outcomes have been described among women, younger adults, and those living alone (Benatti et al., 2020; Fiorillo & Gorwood, 2020; Fiorillo et al., 2020; Giallonardo et al., 2020). Yet only a limited number of longitudinal studies has been reported in the literature. Even more rarely, descriptives of mental correlates before and after COVID-19 have been offered (Castellini et al., 2020, 2021; Meda et al., 2021; Pierce et al., 2021). The present study attempts to overcome some of the limitations in the existing literature, providing a three timepoint analysis of the evolution in the psychopathology in a population of Italian women.

As the perceived self-efficacy is one of the strongest predictors of general distress following a natural disaster (Benight et al., 1999), the authors hypothesized that young adults may have experienced worsened and adverse mental states during the pandemic. For young adults, the lack of possibility to acquire substantial independence during COVID-19 was hypothesized to be correlated with higher mental distress in the younger population as financial hardships undermined economic autonomy and career progressions (Gruber et al., 2021).

In the present study, an additional focus was placed on gender, as women in general and young women in particular seem to have a higher reliance on social-oriented coping strategies (Mélendez et al., 2012; Tamres et al., 2002). Interestingly, social-oriented coping strategies seem to be beneficial in the short-term, but less effective in the medium or long-term (Stanton & Franz, 1999). Thus, the working hypothesis was to observe worse outcomes at subsequent follow-ups in women, in accordance with previous literature (Rogowska et al., 2021).

The present study also aimed at estimating the role of other significant moderators, as supported by previous research. For instance, insecure attachment styles have been consistently associated to the onset of mental disorders or their worse outcome (Gumley et al., 2014; Pasalich et al., 2012). Adult attachment styles can be defined as patterns of interpersonal behaviors and relational expectations that originate from the internalization of the quality of the interactions with caregivers during infancy (Fraley & Shaver, 2000; Shaver & Mikulincer, 2002). When these interactions are negative, the individual may develop a so-called insecure attachment style, which is characterized by high levels of attachment anxiety or avoidance (Bowlby, 1973; Mikulincer & Shaver, 2012). Attachment anxiety is characterized by a constantly activated state of vigilance, as a precautionary measure foreseeing the separation from partners or caregivers (Fraley et al., 2006), whereas attachment avoidance is associated to the tendency to dismiss otherwise desired emotional connections and strives for self-reliance (Carvallo & Gabriel, 2006; Norris et al., 2012). In contrast, secure attachment style is posited to result from the internalization of positive maternal/paternal models and is characterized by positive interpersonal expectations and functional emotion regulation strategies (Mikulincer & Shaver, 2007, 2012). Relevantly, attachment patterns have been observed to moderate relationship functioning during COVID-19 quarantines, suggesting an erosion in success for social-oriented individual coping strategies (Overall et al., 2022).

For what concerns early negative events, childhood traumatic experiences have also been found to consistently moderate subjective responses to stressful circumstances (Maschi et al., 2013; Nöthling et al., 2020). Lockdowns, lack of access to essential services, and social isolation were all considered potentially significant stressors for traumatized individuals, as previous research showed a heightened tendency to react to stress among victims of abuse (Zhai et al., 2019). As attachment patterns and childhood traumatic experiences are among the most relevant factors explaining both fragility and resilience in response to mental distress (Hailes et al., 2019; Kuo et al., 2015; McKay et al., 2021), the authors analyzed their role in the development of unique longitudinal trajectories during the COVID-19 pandemic.

### Aims

The present study aims at evaluating the differential impact of initial and prolonged COVID-19 pandemic in women, using a three time-point design (before pandemic, during first lockdown, after prolonged time). The primary endpoint was to report longitudinal outcome of psychopathology from pre-pandemic period to subsequent assessments during the COVID-19 pandemic. Secondary endpoints were to quantify the moderation role of age, as well as adult attachment styles and childhood traumatic experiences.

## Materials and Methods

This study has been performed in accordance with the ethical standards as laid down in the 1964 Declaration of Helsinki and its later amendments. This study was approved by the local Ethics Committee. The protocol was firstly approved on the 8th of October 2019, as part of a general assessment of psychopathology in a control group (registration number CEAVC 14,655). Later amendments, due to the insurgence of the COVID-19 pandemic, were approved on the 14th of April of 2020 (registration number CEAVC 17063). All participants have given informed consent for participation in the research study.

### Study design

This observational, longitudinal study involved subjects recruited in the Italian women population a few weeks before the first cases of COVID-19 in Italy (T0), who were then re-evaluated after the Italian Government declaration of lockdown (T1) (Decreto-Legge 9 Marzo 2020, n. 14, 2020) and after prolonged conditions of lockdown, insecurity, and persistence of COVID-19 (T2).

### Participants

The sample was composed by a control group of women that was undergoing an assessment of general psychopathology in other studies by the same research group. All women were asked to provide consent for their participation. Participants were recruited using convenience and snowball sampling methods, with the following inclusion criteria: age between 18 and 60 years old, Italian nationality, and residency in Tuscany. Exclusion criteria included illiteracy or inability to provide consent or to complete the survey online, not being a woman, not having one evaluation at each timepoint.

### Instruments

The Brief Symptom Inventory (BSI; Derogatis & Melisaratos, 1983) is a self-reported 53-items questionnaire aimed at quantifying general psychopathological symptoms in adults over the previous 7 days. It provides a total score, Global Severity Index (GSI; Kellett et al., 2003), and 9 subscales: Somatization (Cronbach’s alpha 0.80, test–retest reliability 0.68), Obsession-Compulsion (Cronbach’s alpha 0.83, test–retest reliability 0.85), Interpersonal Sensitivity (Cronbach’s alpha 0.74, test–retest reliability 0.85), Depression (Cronbach’s alpha 0.85, test–retest reliability 0.84), Anxiety (Cronbach’s alpha 0.81, test–retest reliability 0.79), Hostility (Cronbach’s alpha 0.78, test–retest reliability 0.81), Phobic Anxiety (Cronbach’s alpha 0.77, test–retest reliability 0.91), Paranoid Ideation (Cronbach’s alpha 0.77, test–retest reliability 0.79), and Psychoticism (Cronbach’s alpha 0.71, test–retest reliability 0.78).

The Childhood Trauma Questionnaire-Short Form (CTQ; Bernstein et al., 2003) is a self-reported 28-items questionnaire aimed at measuring exposure to early trauma during childhood retrospectively among adults. The questionnaire measures exposure to early trauma through one total score and five domains (Sacchi et al., 2018): emotional neglect (ordinal alpha 0.91), emotional abuse (ordinal alpha 0.88), sexual abuse (ordinal alpha 0.96), physical neglect (ordinal alpha 0.87), and physical abuse (ordinal alpha 0.95). Higher scores represent higher exposure to early trauma.

Experiences in Close Relationships Questionnaire-Revised (ECR; Fraley et al., 2011) is a 36-items self-reported questionnaire aimed at evaluating the romantic attachment pattern in adult relationships. It quantifies two dimensions (18 items per domain), namely anxious (Cronbach’s alpha 0.88) and avoidant attachment (Cronbach’s alpha 0.79, Busonera et al., 2014).

### Assessment

Three evaluations through self-assessment were administered. T0 was originally collected between the 1st of December 2019 and the 15th of January 2020 (45 days), as part of an observational study on the mental health status in the Tuscan general population. T1 was collected from the 22nd of April 2020 to the 3rd of June 2020 (42 days). Finally, T2 was collected between the 19th of October 2020 and the 20th of December 2020 (62 days). The last timepoint was collected over a longer period of time as to enhance completion. Sociodemographic data were collected at each time-point (age, sex, occupation, and educational level).

The questionnaires did not have an option to opt-out from specific questions or parts of the survey. All questions had to be filled before continuing, and all questions needed to be filled in order to submit the results. All evaluations, at any timepoint, included the following self-administered questionnaire: BSI (Derogatis & Melisaratos, 1983).

In order to evaluate the moderation effect of childhood traumatic experiences and abuse on the longitudinal trajectories, CTQ (Bernstein et al., 2003) was administered at T0. In order to evaluate the moderation effect of attachment patterns in light of the effect of stressors, ECR (Fraley et al., 2011) was administered at T0 as well.

### Statistical analyses

In order to evaluate the impact of initial and prolonged conditions of insecurity due to COVID-19, a repeated measures approach was adopted for longitudinal analyses, using a Linear Mixed Model with random intercepts for each participant and time as a fixed effect.

The psychopathological domains of the CTQ questionnaire were evaluated for their moderation role in the longitudinal analyses. As to control for the role of attachment patterns in the longitudinal evolution of psychopathology of women before and during the COVID-19 pandemic, the Anxiety and Avoidance dimensions of the ECR questionnaire were evaluated for their moderation role.

### Additional Moderation Analyses

Additional analyses were carried forward, in accordance to previous literature on the risk factors for worsening mental status during the pandemic in the Italian general population (Delmastro & Zamariola, 2020; Rossi et al., 2020; Xiong et al., 2020). As to exclude the role of specific social determinants in the sample, linear mixed models were estimated. The moderation role of being a healthcare worker was evaluated, as well as having a bachelor’s degree (or higher) as education. As employment and work are main facets of daily life, and more so under conditions of lockdown, the moderation role of going out of the house for work was analyzed.

## Results

### Descriptive Statistics of the Sample

Three hundred three individual observations were collected in the longitudinal observations, representing 101 unique participants, each with one evaluation at each timepoint. The average age at T0 was 30 years old, with a standard deviation of 10.7. The proportion of healthcare workers in the sample was stable at all the three timepoints (meaning most healthcare workers continued working in the same field at all timepoints), and it was represented by 21 individuals (20.79%). The proportion of women having a partner or living with a partner was also stable at all three time points (72.28% and 29.70%, respectively, meaning most women did not change relationship status during the pandemic). The sample was composed by only women, and the majority of the sample was representative of a well-educated population (63.37% with a college degree at T0).

To be noted, the prevalence of having/having had a loved person infected with COVID-19 rapidly increased from T0 to T2 (from below 9 to 26.67%, Fisher exact test *p* < 0.01). The prevalence of having had a loved one hospitalized for COVID-19 or having had a loved one die for COVID, increased at T2 as well (from 1.98 to 8.89% for hospitalization, 0.99 to 2.22% for death).

### Differential Effect of Initial and Prolonged Conditions of Pandemics

**Fig. 1:**
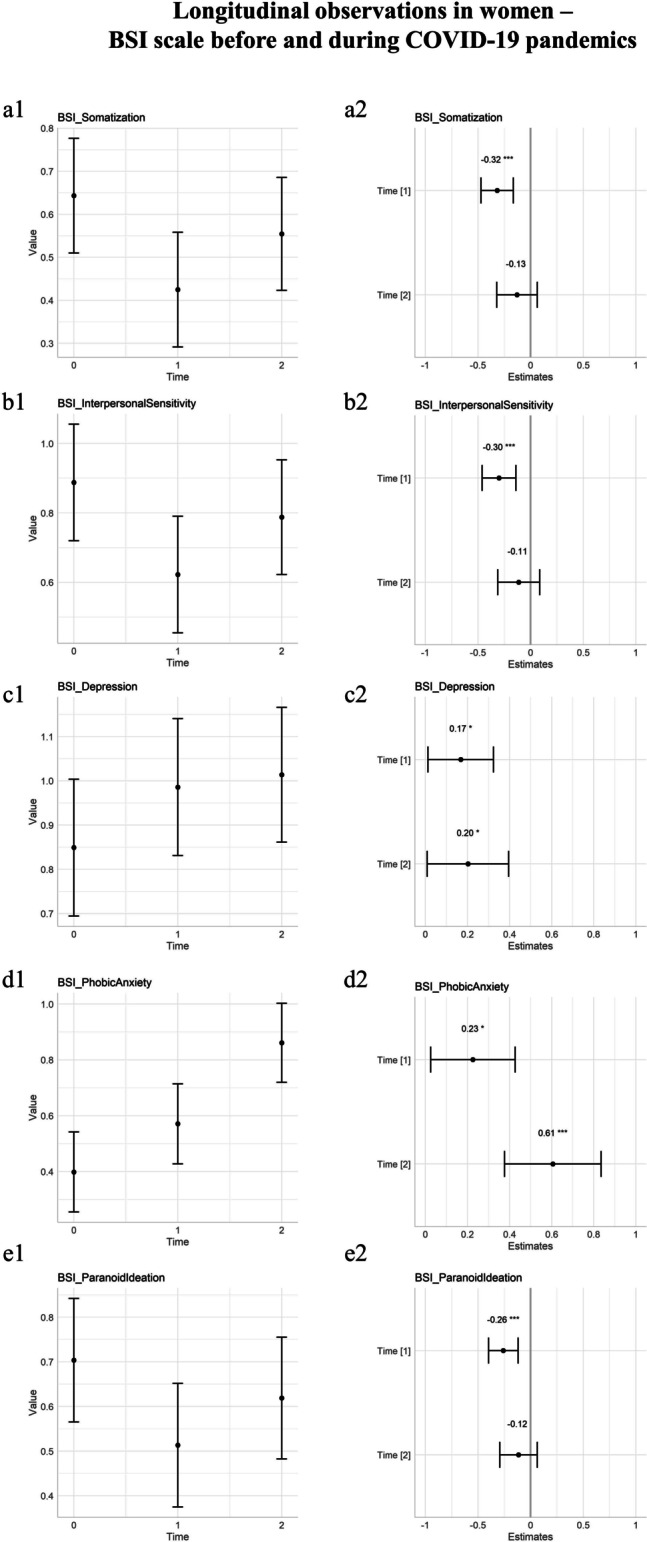
Longitudinal observations of psychopathology in women before and during the COVID-19 pandemic, BSI scale. T0 before COVID-19 lockdowns, T1 during initial lockdowns, T2 after prolonged conditions of pandemics. On the first column, longitudinal observations were plotted as a function of time with an estimate for standard deviations. On the second column, estimates of time coefficients were plotted and associated *p* values annotated. The estimates of coefficients in the second columns are standardized by dividing them by two standard deviations. **a** Somatization domain of BSI. **b** Interpersonal Sensitivity domain of BSI. **c** Depression domain of BSI. **d** Phobic Anxiety domain of BSI. **e** Paranoid Ideation domain of BSI. Note: Brief Symptom Inventory (BSI), significance codes: “***” 0.001; “**” 0.01; “*” 0.05

Multiple linear mixed models were calculated, with the effect of time as a fixed effect. The linear mixed models were modeled to predict the beta coefficients of T1 and T2 in respect to the baseline at T0. Beta coefficients for the fixed effect of time can be found in found in the text as Table 1 for BSI. Statistically significant scales for longitudinal observations were thus plotted in Fig. 1. In particular, the Somatization, the Interpersonal Sensitivity, and the Paranoid Ideation domains of BSI had an initial decline followed by a return to the mean of T0. On the contrary, the Depression domain of BSI showed a concordant trajectory at T1 and T2, with an initial increase followed by a stabilization on higher values in respect to the baseline. The Phobic Anxiety domain showed an initial increase in respect to the baseline at T1, elevated also at T2.

**Table 1:** Differential effect of initial and prolonged conditions of pandemics: BSI

Domain	Estimate intercept	Intercept (*p*)	Estimate T1 (initial lockdown)	T1 (*p*)	Estimate T2 (prolonged pandemics)	T2 (*p*)
Somatization	**0.643** **(± 0.068)**	< 0.001 *	** − 0.218** **(± 0.054)**	< 0.001 *	** − **0.089 (± 0.068)	0.19
Obsession-compulsion	**0.868** **(± 0.082)**	< 0.001 *	** − **0.038 (± 0.069)	0.582	0.027 (± 0.085)	0.75
Interpersonal sensitivity	**0.888** **(± 0.086)**	< 0.001 *	** − 0.265** **(± 0.072)**	< 0.001 *	** − **0.100 (± 0.089)	0.264
Depression	**0.849** **(± 0.079)**	< 0.001 *	**0.137** **(± 0.065)**	0.036 *	**0.165** **(± 0.081)**	0.042 *
Anxiety	**0.938** **(± 0.081)**	< 0.001 *	** − **0.065 (± 0.068)	0.343	0.082 (± 0.084)	0.335
Hostility	**0.607** **(± 0.064)**	< 0.001 *	** − **0.024 (± 0.059)	0.685	0.080 (± 0.071)	0.256
Phobic anxiety	**0.399** **(± 0.073)**	< 0.001 *	**0.172** **(± 0.078)**	0.030 *	**0.462** **(± 0.089)**	< 0.001 *
Paranoid ideation	**0.703** **(± 0.071)**	< 0.001 *	** − 0.190** **(± 0.052)**	< 0.001 *	** − **0.085 (± 0.067)	0.207
Psychoticism	**0.603** **(± 0.0067)**	< 0.001 *	** − **0.051 (± 0.052)	0.330	0.033 (± 0.066)	0.618
Global severity index	**0.765** **(± 0.063)**	< 0.001 *	** − **0.071 (± 0.044)	0.108	0.004 (± 0.057)	0.939

Bold and * for significant results, Brief Symptom Inventory (BSI)

± Standard error

### Moderation Effect of Age

As to evaluate the moderation effect of age, linear mixed models were built using time, age, and time × age as fixed effects. Individual intercepts at T0 were allowed to be calculated.

As presented in Table 2, the interaction of age was significant at T1, during initial lockdown, for the Somatization, Obsession-Compulsion, Interpersonal Sensitivity, Depression, Paranoid Ideation, and Global Severity Index domains. Increased age was protective against the severity index of psychopathology also at T2 for the Somatization and the Interpersonal Sensitivity domains.

**Table 2:** Differential effect of initial and prolonged conditions of pandemics: the moderation role of age on BSI

Domain	Estimate T1: Age (initial lockdown)	T1: Age (*p*)	Estimate T2: Age (prolonged pandemics)	T2: Age (*p*)
Somatization	** − 0.012** **(± 0.005)**	0.015 *	** − 0.013** **(± 0.007)**	0.050 *
Obsession-compulsion	** − 0.016** **(± 0.006)**	0.008 *	** − **0.011 (± 0.009)	0.230
Interpersonal sensitivity	** − 0.016** **(± 0.006)**	0.016 *	** − 0.019** **(± 0.009)**	0.042 *
Depression	** − 0.017** **(± 0.006)**	0.004 *	** − **0.011 (± 0.008)	0.180
Anxiety	** − **0.008 (± 0.006)	0.216	** − **0.009 (± 0.009)	0.318
Hostility	** − **0.009 (± 0.005)	0.112	** − **0.008 (± 0.008)	0.299
Phobic anxiety	0.013 (± 0.007)	0.054	** − **0.004 (± 0.009)	0.673
Paranoid ideation	** − 0.010** **(± 0.005)**	0.038 *	** − **0.011 (± 0.007)	0.106
Psychoticism	** − **0.003 (± 0.005)	0.566	0.001 (± 0.007)	0.916
Global severity index	** − 0.009** **(± 0.004)**	0.026 *	** − **0.001 (± 0.006)	0.085

Bold and * for significant results, Brief Symptom Inventory (BSI)

± Standard error

**Fig. 2:**
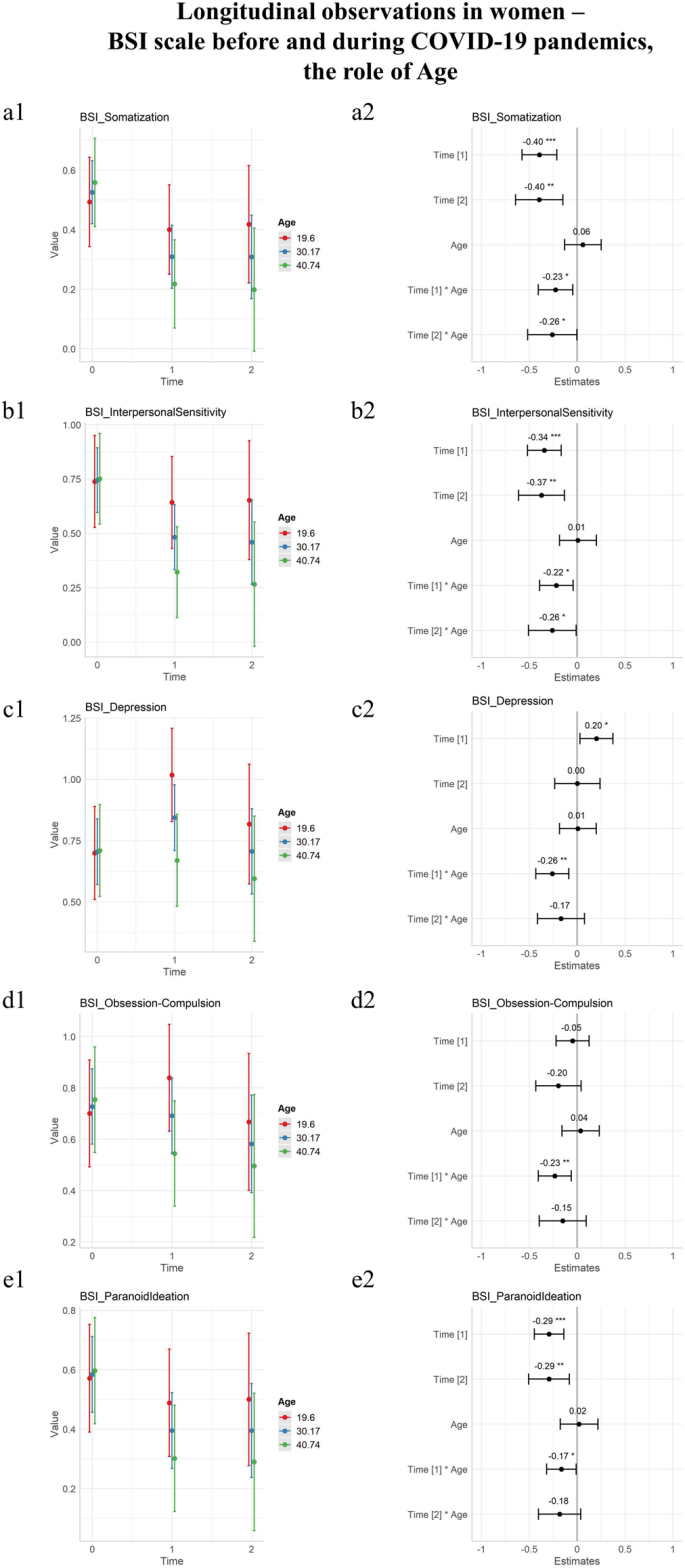
Longitudinal observations of psychopathology in women before and during the COVID-19 pandemic, BSI scale including the moderation role of age. T0 before COVID-19 lockdowns, T1 during initial lockdowns, T2 after prolonged conditions of pandemics. On the first column, longitudinal observations were plotted as a function of time with an estimate for standard deviations. On the second column, estimates of time coefficients were plotted and associated *p* values annotated. The estimates of coefficients in the second columns are standardized by dividing them by two standard deviations. Three colors represent age classes, blue for the mean age of the group, plus or minus a standard deviation (red and green, respectively). **a** Somatization domain of BSI. **b** Interpersonal Sensitivity domain of BSI. **c** Depression domain of BSI. **d** Obsession-Compulsion domain of BSI. **e** Paranoid Ideation domain of BSI. Note: Brief Symptom Inventory (BSI), significance codes: “***” 0.001; “**” 0.01; “*” 0.05

The statistically significant results were plotted in Fig. 2. For all statistically significant interactions, increased age was protective.

### Moderation Effect of Attachment Patterns

The attachment patterns of Anxiety and Avoidance were evaluated. The domains obtained from the ECR questionnaire were evaluated in a dimensional manner, rather than categorical.

The Anxiety dimension of the ECR had a significant effect as a fixed effect for the Somatization (*p* 0.024), Obsession-Compulsion (*p* 0.003), Interpersonal Sensitivity (*p* < 0.001), Depression (*p* < 0.001). Anxiety (*p* 0.010), Paranoid Ideation (*p* 0.006), and the Psychoticism (*p* < 0.001) domains of the BSI, as well as the Global Severity Index (*p* < 0.001). No statistically significant interaction with time was found at T1, while at T2, the statistically significant interactions were with all domains with the exclusion of the Depression and Psychoticism domains. Results were reported in Table 3.

**Table 3:** Differential effect of initial and prolonged conditions of pandemics: the moderation role of the anxiety attachment style on BSI

Domain	Estimate T1: ECR_Anxiety (initial lockdown)	T1: ECR_Anxiety (*p*)	Estimate T2: ECR_Anxiety (prolonged pandemics)	T2: ECR_Anxiety (*p*)
Somatization	0.005 (± 0.004)	0.142	**0.011** **(± 0.004)**	0.002 *
Obsession-compulsion	0.005 (± 0.005)	0.326	**0.011** **(± 0.005)**	0.021 *
Interpersonal sensitivity	− 0.006 (± 0.005)	0.232	**0.010** **(± 0.005)**	0.043 *
Depression	0.002 (± 0.005)	0.737	0.006 (± 0.005)	0.209
Anxiety	− 0.002 (± 0.005)	0.723	**0.010** **(± 0.004)**	0.025 *
Hostility	0.007 (± 0.004)	0.097	**0.017** **(± 0.004)**	< 0.001 *
Phobic anxiety	0.004 (± 0.006)	0.470	**0.016** **(± 0.005)**	0.003 *
Paranoid ideation	− 0.001 (± 0.004)	0.909	**0.010** **(± 0.003)**	0.009 *
Psychoticism	-0.004 (± 0.004)	0.299	0.004 (± 0.003)	0.283
Global severity index	0.001 (± 0.002)	0.768	**0.008** **(± 0.002)**	0.005 *

Bold and * for significant results, Brief Symptom Inventory (BSI)

± Standard error

The Avoidance dimension had a significant effect for Obsession-Compulsion (*p* 0.001), Interpersonal Sensitivity (*p* 0.039), Depression (*p* 0.003), Anxiety (*p* < 0.001), Paranoid Ideation (*p* 0.020), Psychoticism (*p* < 0.001), and Global Severity Index (*p* < 0.001) as a fixed effect, but no statistically significant interaction with time.

**Fig. 3:**
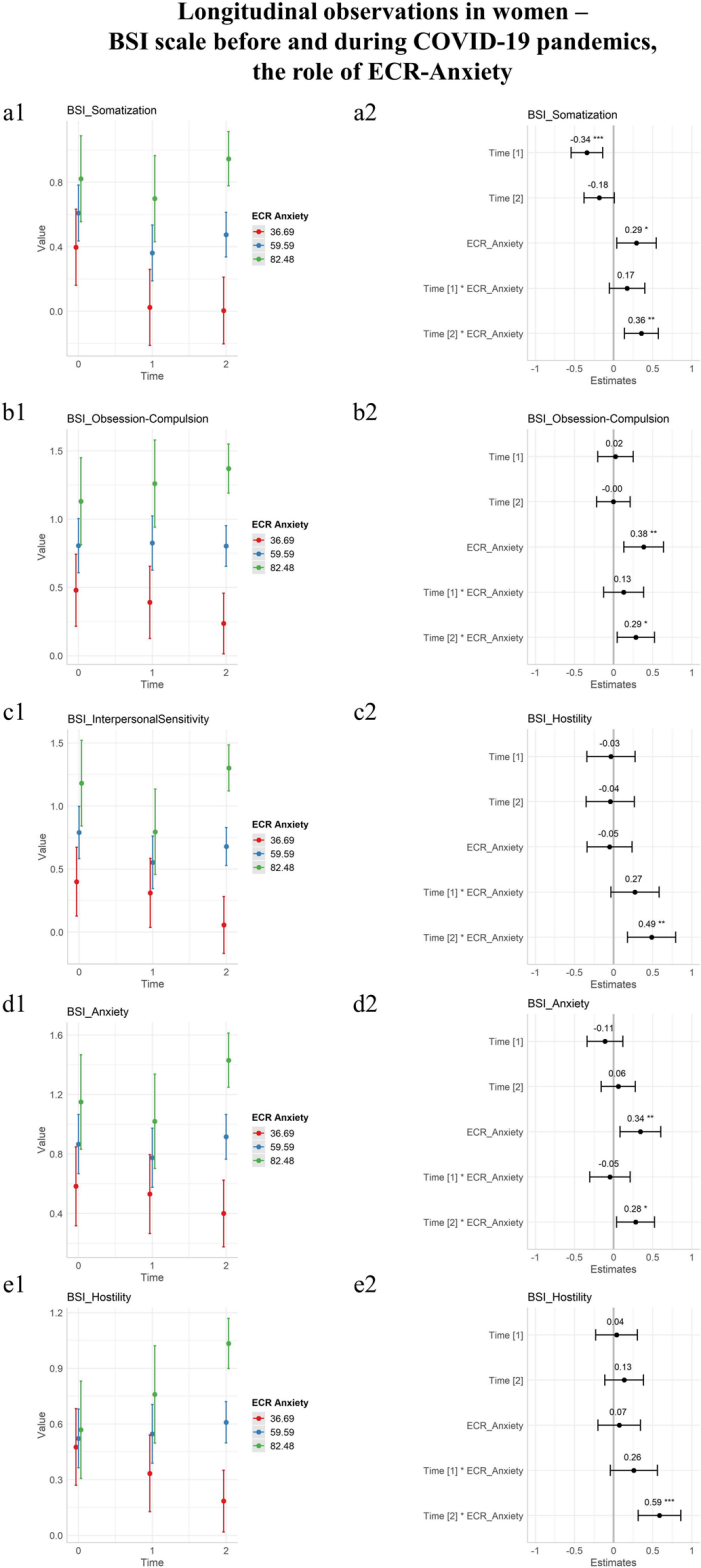

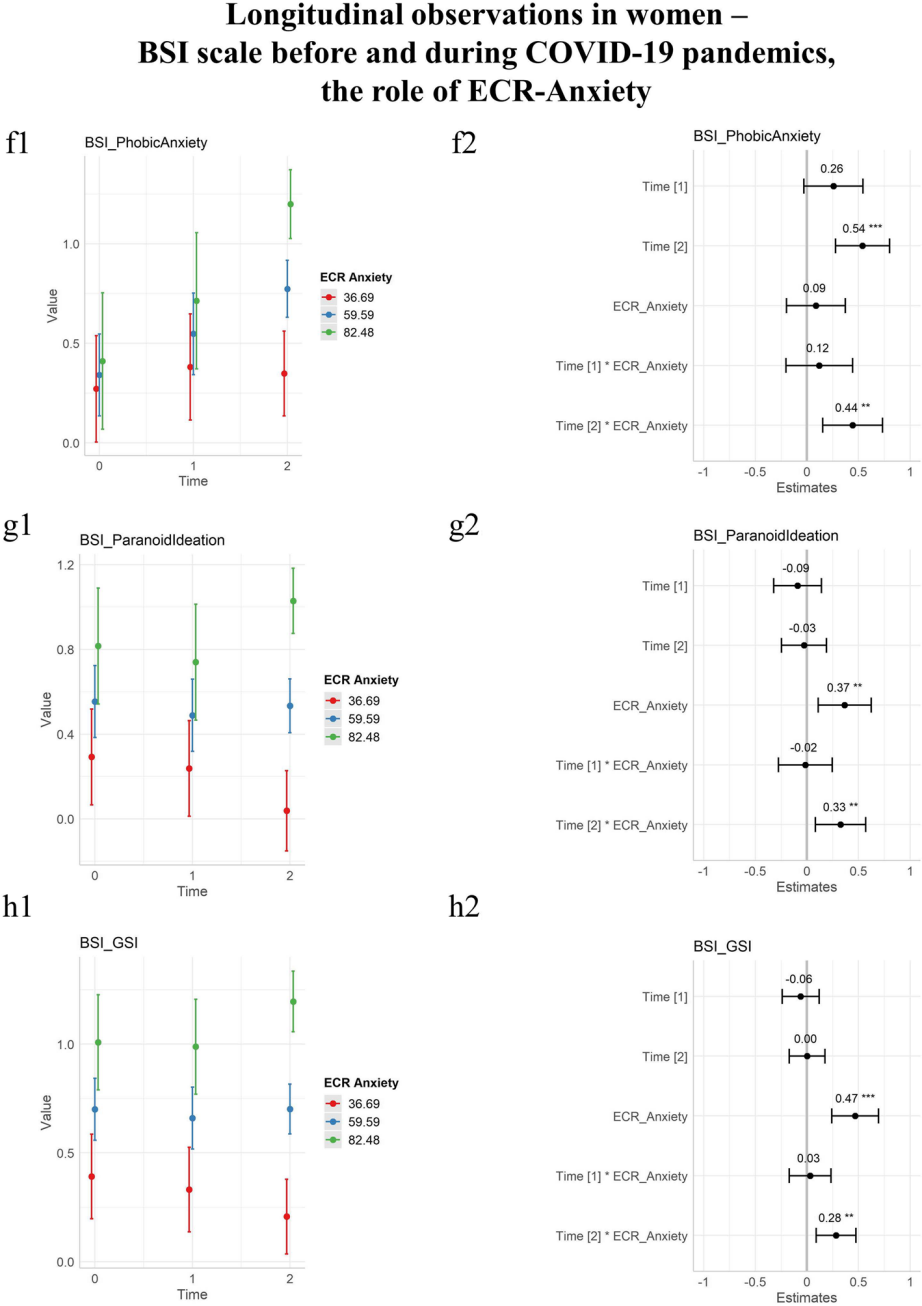
Longitudinal observations of psychopathology in women before and during the COVID-19 pandemic, BSI scale including the moderation role of the Anxiety Pattern of Adult Attachment. T0 before COVID-19 lockdowns, T1 during initial lockdowns, T2 after prolonged conditions of pandemics. On the first column, longitudinal observations were plotted as a function of time with an estimate for standard deviations. On the second column, estimates of time coefficients were plotted and associated *p* values annotated. The estimates of coefficients in the second columns are standardized by dividing them by two standard deviations. Three colors represent age classes, blue for the mean anxiety score of the group, plus or minus a standard deviation (red and green, respectively). **a** Somatization domain of BSI. **b** Obsession-Compulsion domain of BSI. **c** Interpersonal Sensitivity domain of BSI. **d** Anxiety domain of BSI. **e** Hostility domain of BSI. **f** Phobic Anxiety domain of BSI. **g** Paranoid Ideation domain of BSI. **h** Global Severity Index domain of BSI. Note: Brief Symptom Inventory (BSI), significance codes: “***” 0.001; “**” 0.01; “*” 0.05

The statistically significant results were plotted in Fig. 3. For all statistically significant interactions, the Anxiety style of attachment was detrimental.

### Moderation Effect of Childhood Traumatic Experiences

The domains of the CTQ questionnaire were evaluated for their moderation role in the longitudinal analyses. All domains, apart from Sexual Abuse and Physical Neglect, had a significant role as a fixed effect for the Depression (Emotional Abuse *p* 0.028, Emotional Neglect *p* 0.012, Physical Abuse *p* 0.034), Hostility (Emotional Abuse *p* 0.001, Emotional Neglect *p* 0.037, Physical Abuse *p* 0.022), Interpersonal Sensitivity (Emotional Abuse *p* < 0.001, Emotional Neglect *p* 0.001, Physical Abuse *p* 0.017), Paranoid Ideation (Emotional Abuse *p* 0.001, Emotional Neglect *p* 0.022, Physical Abuse *p* 0.012), and the Global Severity Index domain (Emotional Abuse *p* < 0.001, Emotional Neglect *p* 0.023, Physical Abuse *p* 0.009).

For Emotional Abuse, a statistically significant fixed effect was also found for the Psychoticism domain (*p* 0.003). Emotional Neglect showed a statistically significant fixed effect for the Obsession-Compulsion (*p* 0.027) and the Psychoticism (*p* 0.048) domains.

For Physical Abuse, a statistically significant fixed effect was found for the Phobic Anxiety (*p* 0.023) and the Somatization (*p* 0.004) domains. Physical Neglect had a single statistically significant fixed effect for the Interpersonal Sensitivity domain (*p* 0.030). Sexual Abuse showed a marked different trend than the other domains of the CTQ questionnaire, as Physical Neglect it had a single statistically significant fixed effect for Interpersonal Sensitivity (*p* 0.037), but also showed significant interactions with time for the Anxiety and Hostility domains.

**Fig. 4:**
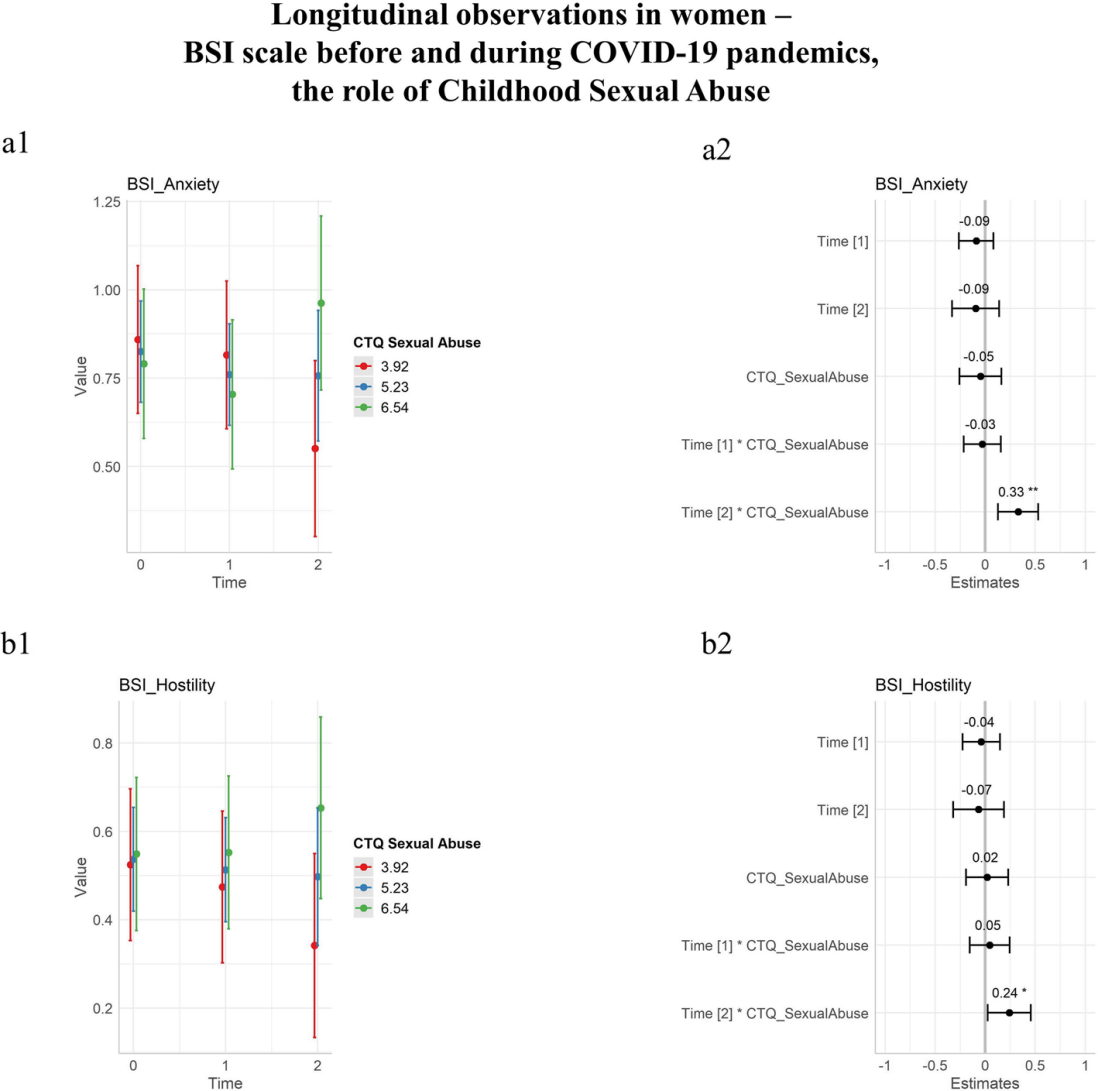
Longitudinal observations of psychopathology in women before and during the COVID-19 pandemic, BSI scale including the moderation role of the Childhood Sexual Abuse. T0 before COVID-19 lockdowns, T1 during initial lockdowns, T2 after prolonged conditions of pandemics. On the first column, longitudinal observations were plotted as a function of time with an estimate for standard deviations. On the second column, estimates of time coefficients were plotted and associated *p* values annotated. The estimates of coefficients in the second columns are standardized by dividing them by two standard deviations. Three colors represent age classes, blue for the mean sexual abuse score of the group, plus or minus a standard deviation (red and green, respectively). **a** Anxiety domain of BSI. **b** Hostility domain of BSI. Note: Brief Symptom Inventory (BSI), significance codes: “***” 0.001; “**” 0.01; “*” 0.05

Results for Sexual Abuse interaction with time were plotted in Fig. 4 and described in Table 4.

**Table 4:** Differential effect of initial and prolonged conditions of pandemics: the moderation role of childhood sexual abuse on BSI

Domain	Estimate T1: CTQ_Sexual abuse (initial lockdown)	T1: CTQ_Sexual abuse (*p*)	Estimate T2: CTQ_Sexual abuse (prolonged pandemics)	T2: CTQ_Sexual abuse (*p*)
Anxiety	− 0.016 (± 0.005)	0.755	**0.184** **(± 0.006)**	0.002*
Hostility	0.021 (± 0.046)	0.656	**0.110** **(± 0.050)**	0.031*

Bold and * for significant results, Brief Symptom Inventory (BSI)

± Standard error

### Moderation Effect of Social Determinants

The moderation role of being a healthcare worker was evaluated, as well as having a bachelor’s degree or higher as education. No effect of education was statistically significant nor its interaction with time. The moderation role of going out of the house for work was analyzed. No significant effect was found for going out of the house for work nor its interaction with time.

## Discussion

The current study aimed at evaluating the impact of initial and prolonged conditions of pandemic due to COVID-19 on the mental health status of women. Additionally, the role of individual moderators, according to the scientific literature on the topic, was explored. The present study offers rare descriptions of contrasts pre- and post-COVID-19, as a sample of women was enrolled in a previous study as a control group between the 1st of December 2019 and the 15th of January 2020. This first time-point can be reasonably used to infer the differential effects of initial and prolonged conditions of pandemics for what concerns the mental well-being of women enrolled in the sample.

Overall, the present study found concordant trajectories at the two follow-ups for most of the domains under investigation. In particular, at the second follow-up (acquired between October and December 2020), increased levels of Phobic Anxiety and Depression were found, signaling a potential long-lasting decay in the mental status of women. In contrast, other significant domains (Paranoid Ideation, Somatization, and Interpersonal Sensitivity) showed an initial worsening at T1, followed by amelioration and regression to baseline levels at T2.

A recent study provided evidence of differential effects before and after the pandemic on the mental health of the general population (Pierce et al., 2021). Although the study comprised a large sample of individuals, questionnaires were not specific for psychopathology. In fact, the General Health Questionnaire, especially in its short 12 questions form, is intended as a screening tool for use in primary care (Gnambs & Staufenbiel, 2018) and does not provide insight into distinct mental constructs. Nevertheless, current results corroborate previous findings (Pierce et al., 2021), as it was shown that a latent class of individuals with deteriorating mental health, or persistent poor mental health, during the pandemic was principally composed of women.

For what concerns individual moderators of distress, attachment theory has underpinned the research of long-term consequences on mental health, through the analysis of adopted interaction patterns acquired during childhood and adolescence (Prior & Glaser, 2006). Multiple studies confirmed the role of insecure attachment styles as a risk factor for mental disorders (Barnes & Theule, 2019; Dagan et al., 2018; Pallini et al., 2019), and one meta-analysis in particular focused on the role of secure attachment as a core feature for psychological resilience (Darling Rasmussen et al., 2019). Current results confirm that attachment patterns can moderate longitudinal trajectories of psychopathology, especially when considering the higher exposure to relationship conflicts, distance, or hastened cohabitation during the COVID-19 pandemic (Overall et al., 2022; Pietromonaco & Overall, 2021). In fact, periods of quarantine or prolonged lockdowns might have stressed anxious individuals imposing protracted extents of time separated by partners or caregivers (Gruber et al., 2021). As women were previously observed as more likely to adopt social-oriented coping strategies in comparison to males (Mélendez et al., 2012; Tamres et al., 2002), longer isolation periods may have interfered with the successful management of harmful stress in this population. Furthermore, the coping mechanisms of individuals characterized by avoidant attachment styles may have been taxed by sustained and hastened co-habitation (Gruber et al., 2021).

Secondarily, the role of early traumatic experiences during childhood was explored. Although a wide range of studies suggested emotional abuse as a consistent risk factor for diminished mental resilience (Christ et al., 2019; Dye, 2020; Glaser, 2002; Norman et al., 2012), the present study focused on the longitudinal evolution of general psychopathological domains during a time of worldwide natural disaster and its results were not easily predictable by previously published research articles. What the authors found was that emotional abuse could be accounted as a fixed factor at baseline, as emotionally abused women exhibited increased scores of mental distress at the first evaluation. However, emotional abuse was not observed to significantly moderate differential trajectories in time. Other forms of traumatic experiences (either active as physical abuse, or passive as physical or emotional neglect) were observed as fixed effects for differences between women at baseline, but similarly they also did not show a moderating role for trajectories in time. On the contrary, unwanted sexual experiences during childhood had a significant role for the psychopathology of women both as a fixed effect and as a moderating factor. In fact, increased levels of mental distress were found among sexually abused women at the first evaluation, and differential trajectories through time (worse outcome) were observed for what concerns Anxiety and Hostility. The results of the present study seem to confirm previous literature on the topic, which consistently found worse outcomes for sexually abused women rather than other forms of trauma (Chen et al., 2010; Hailes et al., 2019; Lindert et al., 2014; Maniglio, 2013).

In conclusion, other moderators supported by previous literature were evaluated. Higher educational status was not significantly correlated with mental distress levels at baseline, and it did not moderate distress trajectories through time. Similarly, healthcare workers were not observed as exhibiting significantly different levels of distress either at baseline or subsequent follow-ups, in comparison to the other participants. Comparably, individuals working from home did not show significantly different levels of distress at baseline, and differential trajectories in mental health through time were also not observed in comparison to other participants.

### Limitations

T0 was collected between the 1st of December 2019 and the 15th of January 2020, when COVID-19 was already known to have spread rapidly outside of China, the authors would then caution when considering T0 to be partially influenced by an initial impact on the mental health reported in the sample. The authors’ opinion is that the estimated effect on the general psychopathology was therefore biased towards a more neutral approximation. T2 was collected over a wider timespan as compared to T1 in order to enhance completion. The sample size was small, and mostly representative of a highly educated population, thus cautioning generalization to the broader community.

## Conclusions

The present study confirms that Italian women experienced a high burden of distress during COVID-19. Attachment insecurity and traumatic experiences were found to have a significant effect in the interplay between stress and stressors in the sample. Additional individual moderators were evaluated — educational status, being a healthcare worker, work from home — and none of these social determinants resulted significant for explaining longitudinal psychopathological trajectories. The clinical utility of the present study lies in the evidence of the role of attachment patterns and childhood traumatic experiences in the development and maintenance of mental distress during the COVID-19 pandemic. This evidence may warrant a future focus in the clinical setting for the collection of accurate personal history components, in order to better evaluate individual risk factors and to provide adequate treatment interventions.

## Data Availability

The data that support the findings of this study are available from the corresponding author, G.C., upon reasonable request.
